# Nanotechnology for Electronic Materials and Devices

**DOI:** 10.3390/nano12193319

**Published:** 2022-09-23

**Authors:** Raffaella Lo Nigro, Patrick Fiorenza, Béla Pécz, Jens Eriksson

**Affiliations:** 1 Consiglio Nazionale della Ricerche (CNR), Istituto per la Microelettronica e Microsistemi (IMM), Strada VIII, 5, 95121 Catania, Italy; 2 Centre for Energy Research, Institute for Technical Physics and Materials Science Research, Konkoly-Thege, 29-33, 1121 Budapest, Hungary; 3 Department of Physics, Chemistry and Biology (IFM), University of Linkoping, Campus Valla, Fysikhuset, SE-581 83 Linkoping, Sweden

The historical scaling down of electronics devices is no longer the main goal of the International Roadmap for Devices and Systems [1], but the integration of electronic components at the nanoscale is an emerging focus, coupled with the need for novel nanomaterials, emerging characterization methods and device fabrication techniques at the nanoscale. The growing interest in nanomaterials [2] can be associated with their unique properties, which are not present in bulk or thick films, and they are currently finding rapid application in many technological areas (such as high-frequency electronics [3], power devices [4], displays, energy conversion systems, energy storage [5], photovoltaics and sensors). At the same time, fabrication methods based on novel processes and/or approaches must be developed for the synthesis of the nanostructured materials, as well as accurate characterization techniques at the nanoscale for the materials and their interfaces.

In this context, the aim of this Special Issue, entitled “**Nanotechnology for Electronic Materials and Devices**”, is to collect dedicated papers in several nanotechnological fields. The issue consists of eleven selected regular papers focusing on the latest developments in nanomaterials and nanotechnologies for electronic devices and sensors. Thus, topics such as approaches to synthesis, advanced characterization methods and device fabrication techniques have been covered in the present issue.

We editors are aware that, due to the many topics related to the use of nanotechnology for electronics, the present issue cannot provide a comprehensive presentation of the arguments; however, we are confident that the main general areas have been discussed and can be summarized in the following research topics:

(i) Nanoscaled materials and their properties: several papers in this issue focus on nanolaminated oxide combinations (such as hafnium oxide/iron oxide [6] or tungsten oxide/molybdenum oxides [7]) as well as on 2D materials (molybdenum disulphides and its rare-earth-doped-thin layers [8]).
(ii) Moreover, innovative synthesis approaches have been described in some papers for nanomaterials and thin films, such as aluminium nitrides [9], silicon whiskers [10] or vanadium-oxide-rich layers [11].
(iii) Other paper focus on advanced nanoscale characterization, mainly based on scanning-probe methods (scanning non linear dielectric microscopy [12] and high-resolution scanning capacitance spectroscopy [13]), as well as on surface optical techniques (photoluminescence and spectroscopic ellipsometry) [14].
(iv) Finally, some papers are dedicated to device performances [15] and circuit analysis [16], providing evidence that it is crucial to move from research to technological development to control the quality of innovative products and functionalities.

We editors are grateful to all the authors for submitting their scientific results to the present Special Issue and we hope that *Nanomaterials* readership will find interesting imputs in the wider scenario of electronical applications of nanotechnology.

## References

[1] International Roadmap for Devices and Systems (IRDS™) 2021 Edition. https://irds.ieee.org/editions/2021/executive-summary.

[2] Bytler S.Z., Hollen S.M., Cao L.Y., Cui Y., Gupta J.A., Gutierrez H.R., Heinz T.F., Hong S.S., Huang J.X., Ismach A.F. (2013). Progress, Challenges, and Opportunities in Two-Dimensional Materials Beyond Graphene. ACS Nano.

[3] Lo Nigro R., Fiorenza P., Greco G., Schilirò E., Roccaforte F. (2022). Structural and Insulating Behaviour of High-Permittivity Binary Oxide Thin Films for Silicon Carbide and Gallium Nitride Electronic Devices. Materials.

[4] Bose B.K. (1989). Power electronics-an emerging technology. IEEE Trans. Ind. Electron..

[5] Koohi-Fayegh S., Rosen M.A. (2020). A review of energy storage types, applications and recent developments. J. Energy Storage.

[6] Kalam K., Otsus M., Kozlova J., Tarre A., Kasikov A., Rammula R., Link J., Stern R., Vinuesa G., Lendinez J.M. (2022). Memory Effects in Nanolaminates of Hafnium and Iron Oxide Films Structured by Atomic Layer Deposition. Nanomaterials.

[7] Fried M., Bogar R., Takacs D., Labadi Z., Horvath Z.E., Zoinai Z. (2022). Investigation of Combinatorial WO_3_-MoO_3_ Mixed Layers by Spectroscopic Ellipsometry Using Different Optical Models. Nanomaterials.

[8] Li S., Tian S., Yao Y., He M., Chen L., Zhang Y., Zhai J. (2021). Gallic Enhanced Electrical Performance of Monolayer MoS_2_ with Rare Earth Element Sm Doping. Nanomaterials.

[9] Schilirò E., Giannazzo F., Di Franco S., Greco G., Fiorenza P., Roccaforte F., Prystawko P., Kruszewski P., Leszczynski M., Cora I. (2021). Highly Homogeneous Current Transport in Ultra-Thin Aluminum Nitride (AlN) Epitaxial Films on Gallium Nitride (GaN) Deposited by Plasma Enhanced Atomic Layer Deposition. Nanomaterials.

[10] Pecz B., Vouroutzis N., Radnoczi G.Z., Frangis N., Stoemenos J. (2021). Structural Characteristics of the Si Whiskers Grown by Ni-Metal-Induced-Lateral-Crystallization. Nanomaterials.

[11] Posa L., Molnar G., Kalas B., Baji Z., Czigany Z., Petrik P., Volk J. (2021). A Rational Fabrication Method for Low Switching-Temperature VO_2_. Nanomaterials.

[12] Yamasue K., Cho Y. (2022). Boxcar Averaging Scanning Nonlinear Dielectric Microscopy. Nanomaterials.

[13] Fiorenza P., Alessandrino M.S., Carbone B., Russo A., Roccaforte F., Giannazzo F. (2021). High-Resolution Two-Dimensional Imaging of the 4H-SiC MOSFET Channel by Scanning Capacitance Microscopy. Nanomaterials.

[14] Panasci S.E., Koos A., Schilirò E., Di Franco S., Greco G., Fiorenza P., Roccaforte F., Agnello S., Cannas M., Gelardi F.M. (2022). Multiscale Investigation of the Structural, Electrical and Photoluminescence Properties of MoS_2_ Obtained by MoO_3_ Sulfurization. Nanomaterials.

[15] Gao Q., Zhang C., Liu P., Hu Y., Yang K., Yi Z., Lui L., Pan X., Zhang Z., Yang J. (2021). Effect of Back-Gate Voltage on the High-Frequency Performance of Dual-Gate MoS_2_Transistors. Nanomaterials.

[16] Park J., Ra C., Lim J., Jeon J. (2022). Device and Circuit Analysis of Double Gate Field Effect Transistor with Mono-Layer WS_2_-Channel at Sub-2 nm Technology Node. Nanomaterials.

